# POFUT2 Mediated Fucosylation of JUP Enhances VEGFA Expression to Promote Angiogenesis in Colorectal Cancer

**DOI:** 10.7150/ijms.113515

**Published:** 2026-01-01

**Authors:** Yanfeng Zou, Yian Yang, Wei Xu, Peiguo Cao, Zheng Li

**Affiliations:** 1 Department of Oncology, Third Xiangya Hospital, Central South University, Changsha 410013, China.; 2 NHC Key Laboratory of Carcinogenesis, Key Laboratory of Carcinogenesis, Chinese Ministry of Health, Cancer Research Institute, Xiangya School of Basic Medical Sciences, Central South University, Changsha, Hunan, China.

**Keywords:** colorectal cancer, glycosyltransferase, POFUT2, JUP, VEGFA, angiogenesis

## Abstract

**Background:** Colorectal cancer (CRC) ranks as the third most common cancer globally and is characterized by a poor prognosis. Abnormal glycosylation, a hallmark of cancer development, influences multiple signaling pathways and contributes to CRC progression. Identifying key glycosyltransferase genes associated with CRC prognosis could provide novel therapeutic targets and improve patient outcomes.

**Methods:** We utilized datasets from The Cancer Genome Atlas (TCGA) to analyze the expression of glycosyltransferase genes in CRC tissues. Lasso regression and COX regression models were employed to identify key glycosyltransferase genes associated with patient prognosis. Angiogenesis assays were performed utilizing tumor-conditioned medium to assess the influence of GDP-fucose protein O-fucosyltransferase 2 (POFUT2) in cancer cells on human umbilical vein endothelial cells (HUVECs).Flag-Immunoprecipitation combining mass spectrometry detection was employed to identify potential interacting proteins with POFUT2.Western blot, immunoprecipitation, and immunohistochemistry were used to assess the interaction between POFUT2 and Junction Plakoglobin (JUP), as well as the correlation between the expression of Vascular endothelial growth factor A (VEGFA) and Platelet Endothelial Cell Adhesion Molecule-1 (CD31).

**Results:** Our analysis revealed that POFUT2 is significantly upregulated in CRC tissues and correlates with poor prognosis. Elevated POFUT2 expression in CRC cells enhances proliferation, invasion, and angiogenic capabilities of HUVECs. Among POFUT2 potential interacting proteins, three proteins were found to be involved in angiogenesis: JUP, HSBP1 (Heat Shock Factor Binding Protein 1), and AGO2 (Argonaute RISC Catalytic Component 2). Specifically, HSBP1 negatively regulates angiogenesis, whereas JUP and AGO2 positively regulate angiogenesis. Our results demonstrated that POFUT2 promotes the protein expression of JUP but does not affect the expression of AGO2. Further investigations revealed that POFUT2 interacts with JUP, upregulates its expression through fucosylation, and subsequently regulates VEGFA levels, thereby enhancing angiogenesis. A significant positive correlation was observed between POFUT2 and the expression levels of JUP, VEGFA, and CD31 in CRC tissues.

**Conclusions:** POFUT2 is identified as a critical glycosyltransferase gene in CRC, closely associated with angiogenic phenotypes and poor prognosis. High POFUT2 expression in CRC regulates JUP fucosylation, increasing JUP and VEGFA levels, which promotes angiogenesis. These findings suggest POFUT2 could serve as a prognostic marker for colorectal cancer, and targeting it may inhibit angiogenesis and aid in treatment.

## Introduction

Colorectal cancer ranks as the third most frequently diagnosed cancer globally and is the second leading cause of cancer-related mortality^1^. The prognosis for localized CRC is relatively favorable, with a 5-year survival rate that can reach 90%, but this drops significantly for patients with advanced metastatic disease, with a 5-year survival rate of only 14%^2^. Unfortunately, many patients are diagnosed with CRC at a stage where distant metastases have already occurred, precluding the possibility of curative surgery^3^. The challenges posed by metastasis and disease recurrence are significant in the clinical management of CRC^4^. Consequently, uncovering novel molecular mechanisms that influence the progression and prognosis of CRC, as well as discovering new therapeutic targets, remain a priority in oncological research 5.

Glycosylation, a prevalent post-translational modification of proteins, is intricately linked to the pathogenesis and progression of cancer 6-8. Throughout tumorigenesis, glycosylation influences a multitude of biological processes, including cell signaling, tumor invasion, proliferation, angiogenesis, metastasis, protein maturation, and turnover^9^-^11^. Glycosylation protein markers, such as CA19-9, CA125, CEA, PSA, and AFP, have been instrumental in clinical early diagnosis and recurrence prediction^12^-^16^. Glycosyltransferases are pivotal in cancer development due to their role in inducing abnormal glycosylation of tumor-associated glycoproteins, thereby impacting tumor progression^9^,^17^-^20^. In breast cancer, the fucosyltransferase FUT8 catalyzes B7H3 core N-glycosylation, maintaining its high expression levels. The combination of 2F-Fuc, an inhibitor of core fucosylation, with anti-PDL1 enhances therapeutic efficacy in B7H3-positive TNBC tumors^21^. The microphthalmia-associated transcription factor (MITF) is activated through O-GlcNAcylation by the O-GlcNAc transferase (OGT), which facilitates its translocation to the nucleus, thereby inhibiting senescence in palbociclib-induced breast cancer drug-resistant cells^22^. In CRC, the depletion of OGT markedly amplifies TCA cycle activity. By facilitating O-GlcNAcylation at c-Myc's serine 415, OGT bolsters c-Myc stability, which in turn transcriptionally elevates PDK2 levels. This enhancement curbs reactive oxygen species production and fosters the proliferation of xenografted tumors^23^.In metastatic colorectal cancer, the FDW028 has demonstrated significant anti-tumor activity by targeting FUT8. Specifically, FDW028 catalyzes the defucosylation of B7-H3, thereby triggering its degradation via the HSC70/LAMP2A pathway^24^. In addition, novel FUT8 inhibitor compound 15 has a significant antitumor effect in a colorectal cancer cell xenograft model, suggesting its great potential in the treatment of colorectal cancer^25^.

To investigate the correlation between the expression of 200 glycosyltransferase-related genes (GTs) and the diagnostic and prognostic implications of CRC, we analyzed the CRC transcriptome data from The Cancer Genome Atlas (TCGA). Lasso regression and multivariate COX regression analyses, we constructed a prediction model including seven GTs: POFUT2, ALG1L2, HAS1, PYGL, COLGALT2, B3GNT4, and GALNT7. Notably, CRC patients with high expression of POFUT2 had a poorer prognosis and POFUT2 exhibited a high AUC of 0.8. Consistent with previous studies that report a significant increase in POFUT2 expression levels in patients with colorectal tumours, their findings suggest that POFUT2 may serve as a potential biomarker and therapeutic target for CRC^26^. We focused POFUT2 function and potential mechanisms in CRC in this study.

POFUT2 is a member of the fucosyltransferase (FUT) family and acts as an O-fucosyltransferase, which is responsible for the direct addition of fucose to serine or threonine residues on glycoproteins^27^. FUTs enzymes catalyze the transfer of fucose residues from GDP-fucose donor substrates to acceptor substrates on oligosaccharides, glycoproteins, and glycolipids^28^. O-fucosylation has been implicated in the secretion of various proteins 29,30. Mice with a knockout of the POFUT2 gene exhibit embryonic lethality due to defects in protozoal embryo formation, highlighting the essential role of POFUT2 in early development^31^. Previous study reported that the expression levels of POFUT2 are significantly elevated in patients with colorectal tumors^32^. Nevertheless, our comprehension of the function and mechanism of action of POFUT2 in CRC remains limited.

Angiogenesis plays a key role in tumor metastasis. It not only provides the necessary nutrients and oxygen for tumor growth, but also creates conditions for tumor cell invasion and distant metastasis^33^,^34^. We utilized the CancerSEA database analysis and found that POFUT2 was highly positively correlated with the angiogenic phenotype. We found that the increased expression of POFUT2 in colorectal cancer cells significantly promoted the angiogenic ability of HUVECS. Furthermore, we identified an interaction between POFUT2 and JUP proteins, with POFUT2 modifying JUP through fucose glycosylation, thereby enhancing JUP's protein expression. Subsequent investigations revealed that POFUT2 increases VEGFA expression by modulating JUP, which in turn drives angiogenesis in CRC. Collectively, our findings suggest that POFUT2 serves as a potential molecular diagnostic marker and a candidate therapeutic target for anti-angiogenic strategies in CRC patients.

## Materials and Methods

### Patients and samples

The study involved 20 pairs of tissue samples and corresponding patient data, which were procured from Central South University. Ethical approval for this research was granted by the Ethical Review Committee of the Third Xiangya Hospital, affiliated with Central South University. Prior to their participation, all patients provided their written informed consent, ensuring compliance with ethical standards and patient autonomy.

### Cell lines and cell culture

We obtained the human immortalized colorectal epithelial cell line NCM460, as well as human CRC cell lines HCT8, LOVO, CACO2, HCT116, and HUVECs from the Cell Center of Central South University. All cell lines were cultured in Dulbecco's Modified Eagle Medium (DMEM) supplemented with 10% fetal bovine serum. The cells were maintained in a controlled environment of a 37℃ incubator with a 5% CO2 atmosphere to ensure optimal growth conditions.

### Cells transfection and tumor conditioned medium (TCM) obtaining

Colorectal cancer cells were transfected with either a pcDNA3.1-3xFlag-POFUT2 overexpression plasmid or siRNA targeting POFUT2. For controls, we used an empty plasmid in the overexpression group and si-NC (non-targeting control siRNA) in the knockdown group. HCT8 and Lovo cells were transiently transfected using the jetPRIME transfection reagent (jetPRIME, polyplus, 101000001). Twelve hours post-transfection, the culture medium was replaced with serum-free medium. TCM were collected 36 hours later. HUVECs were then cultured in the presence of TCM and subjected to a series of functional assays, including CCK-8 proliferation assay, tube formation assay, and Transwell invasion assay. The specific siRNA sequences used were as follows: for POFUT2, (1: F5′-GGAUGAAGAUGAAGGUCAAT-3′; 2: F 5′-GGAUGUACCCAGUCUGGAAT-3′; 3: F 5′-CGACCACUAUGGAGGGAAAT-3′), for JUP, (1: F5′-GCTTCAGACTCAAGTACCCA-3′; 2: F5′- GATCATGCGTAACTACAGTTA-3′).

### CCK-8 proliferation assay

HUVECs were first digested and then seeded into 96-well plates at a density of 1000 cells per well. The culture medium was mixed with TCM in a 1:1 ratio, with a total volume of 100 µL per well. Following this, 10 µL of the Cell Counting Kit-8 (CCK-8, NCM Biotech, C6005) was added to each well. The plates were then returned to the incubator for an additional 2 hours to allow for colorimetric reaction. Subsequently, the absorbance at 450 nm was determined using a microplate reader to assess cell viability.

### Tubule formation assay

We utilized pure Matrigel (Corning, 356234) to coat a 24-well plate. The Matrigel was allowed to polymerize for 3 hours at a constant temperature of 37°C in an incubator. Subsequently, HUVECs were seeded onto the Matrigel-coated wells at a density of 8 × 10^4^ cells per well. The culture medium was then mixed with TCM in a 1:1 ratio, resulting in a final volume of 400 µL per well. The 24-well plate was incubated for an additional 6 hours in a thermostatic incubator. Post-incubation, the cells were examined under a microscope to evaluate their behavior and morphology within the Matrigel matrix.

### Transwell invasion experiment

The substrate gel was diluted with culture medium at a ratio of 1:8. A volume of 20 µL of this diluted gel was added to each well of a 24-well plate, and the plate was then incubated at 37°C to allow the gel to solidify for 3 hours. Following solidification, digested HUVECs were seeded into the wells at a density of 2.5 × 10^4^ cells per well. The cells were cultured in a medium containing 2% fetal bovine serum (FBS) mixed with TCM in a 1:1 ratio, with a total volume of 200 µL per well. To establish a chemotactic gradient, 600 µL of culture medium enriched with 20% FBS was added to the lower chamber of the 24-well plate. The invasive potential of the HUVECs was evaluated after a 48-hour incubation in a controlled-temperature incubator.

### Quantitative real-time RT-PCR

Total cellular RNA was extracted from the samples using the NCM RNA Extraction Kit (NCM Biotech, M5105) as per the manufacturer's guidelines. Subsequently, the RNA was reverse transcribed into complementary DNA (cDNA) using the HiScript II Q RT SuperMix for qPCR (+gDNA Wiper) (Vazyme, R223-01). Following cDNA synthesis, quantitative real-time polymerase chain reaction (qRT-PCR) was conducted with the HiScript II Q RT SuperMix (Vazyme, R222-01) according to the manufacturer's protocol. The specific primer sequences used were as follows: for β-actin, forward (F 5′-ATTCCTATGTGGGCGACGAG-3′ and R 5′-TAGCACAGCCTGGATAGCAA-3′); for POFUT2, (F 5′-GCAGACATCTCAACTCCACG-3′ and R 5′-TCCTTTCTGACGGCATCTG-3′); for JUP, (F 5′-ACCAGCATCCTGCACAACCTCT-3′ and R 5′-GGTGATGGCATAGAACAGGACC-3′); and for VEGFA, (F 5′-CTGCTGTCTTGGGGTGCATTG-3′ and R 5′-TCACCGCCTCGGGCTTGTCACA-3′).

### Western blot

Cells were lysed using RIPA lysis buffer (NCM Biotech, WB3100) on ice. Once complete lysis was achieved, protein concentrations were determined using the BCA protein assay. The protein samples were then boiled to denature them and subjected to SDS-PAGE for separation. Following electrophoresis, the proteins were transferred onto a PVDF membrane. The membrane was blocked in 5% skimmed milk for 1 hour at room temperature before incubation with the respective primary antibodies at 4°C overnight. After primary antibody incubation, the membrane was incubated with the corresponding secondary antibodies for 2 hours at room temperature. The immunoreactive bands were visualized using an ultrasensitive ECL chemiluminescence detection kit (NCM, P10300). The antibodies used in this study were as follows: POFUT2 (ABclonal, A12223), JUP (ABclonal, A0963), VEGFA (ABclonal, A21647; Proteintech, 19003-1-AP), GAPDH (Proteintech, 10494-1-AP), Flag (ABclonal, AE092), and AAL lectin (Vector Labs, B-1395-1).

### Immunohistochemistry (IHC)

Tissue sections were first deparaffinized by baking in a thermostatic oven at 65°C for 2 hours. Subsequently, the sections were deparaffinized by sequential immersion in xylene and graded ethanol solutions (100%, 95%, 85%, 75%, and 0%) for 3 minutes each. Once dewaxing was complete, antigen retrieval was performed using a 10× Tris-EDTA Antigen Retrieval Solution (Coolaber, SL1863). The sections were then permeabilized in phosphate-buffered saline (PBS) containing 0.1% Triton X-100 for 15 minutes and treated with an endogenous peroxidase blocking buffer (Beyotime, P0100B) for 10 minutes. Following this, the sections were incubated with an immunostaining blocking buffer (Beyotime, P0260) for an additional 10 minutes to reduce non-specific binding. The sections were then incubated with the primary antibody overnight at 4°C, using the same primary antibodies as mentioned earlier. After primary antibody incubation, the sections were incubated with biotinylated goat anti-rabbit IgG (Beyotime, A0279) for 2 hours. This was followed by incubation with a DAB staining kit (ZSGB-BIO, ZLI-9018) for 10 minutes to visualize the immunoreactive sites. Finally, the sections were dehydrated, mounted, and the staining intensity and area were subsequently scored using ImageJ software (developed in Maryland, USA). The stained area score was: < 25% stained area was defined as 1 point, 25% < Stained area < 50% was defined as 2 points, 50% < Stained area < 75% was defined as 3 points, and stained area > 75% was defined as 4 points. Staining intensity was scored as: Negative was defined as 0, Low Positive as 1, and Positive as 2. IHC score = Staining area score * staining intensity score.

### Liquid chromatography-tandem mass spectrometry

Protein samples, after processing, were subjected to separation via SDS-PAGE electrophoresis. The resolved gels were subsequently stained with Thomas Brilliant Blue to visualize the protein bands. The bands of interest were carefully excised from the gel and subjected to in-depth proteomic analysis using liquid chromatography-tandem mass spectrometry (LC-MS/MS) at APTBIO.

### Immunoprecipitation and lectin blotting experiment

Cells were lysed using an IP lysis buffer (NCM Biotech, P70100), and protein samples were collected by centrifugation. To each tube, 20 µl of magnetic beads were added for a 3-hour incubation to facilitate protein cross-linking. Afterward, the excess beads were then washed with the appropriate buffer and 5 µl of the corresponding primary antibody was added to each tube. For the IgG control group, 5 µl of IgG antibody (Santa Cruz Biotechnology, SC-66931) was added. The samples were then incubated at 4 °C overnight to allow for antibody binding. Following the incubation, the IP lysis buffer was used to wash the beads eight times, with each wash lasting for 5 minutes. The proteins that were bound to the beads were subsequently eluted and denatured in preparation for Western blot analysis. For the Western blot procedure, the membrane was first blocked to prevent non-specific binding. It was then incubated with AAL lectin (Vector Labs, B-1395-1) at 4 °C overnight. After primary antibody incubation, the membrane was incubated with biotinylated goat anti-rabbit IgG (Beyotime, A0279) for 2 hours. Finally, the membrane was incubated with a DAB staining kit (ZSGB-BIO, ZLI-9018) for 10 minutes to visualize the staining results.

### Statistical analyses

We retrieved RNA-sequencing data from 521 cases of TCGA CRC cohort from the TCGA database (https://portal.gdc.cancer.gov/). This dataset encompassed sequencing data from both cancerous and paired normal tissues across 521 patients, which were utilized for subsequent bioinformatics analysis. Employing R software, we conducted a series of analyses, including differential gene expression, Lasso regression, univariate and multivariate Cox proportional hazards regression, Kaplan-Meier survival curves, and receiver operating characteristic (ROC) curves. For statistical processing of experimental data, we utilized GraphPad Prism version 9.0.0. To ensure reproducibility, RT-PCR, CCK-8 proliferation assays, tubule formation assays, and Transwell invasion experiments were each performed in triplicate. Data are presented as the mean ± standard deviation (SD). For statistical analysis, independent samples t-tests and one-way ANOVA were employed. P-values < 0.05 were considered statistically significant differences (*p < 0.05, **p < 0.01, ***p < 0.001, ****p < 0.0001).

## Results

### Identify key glycosyltransferases in colorectal cancer

**Figure 1:**
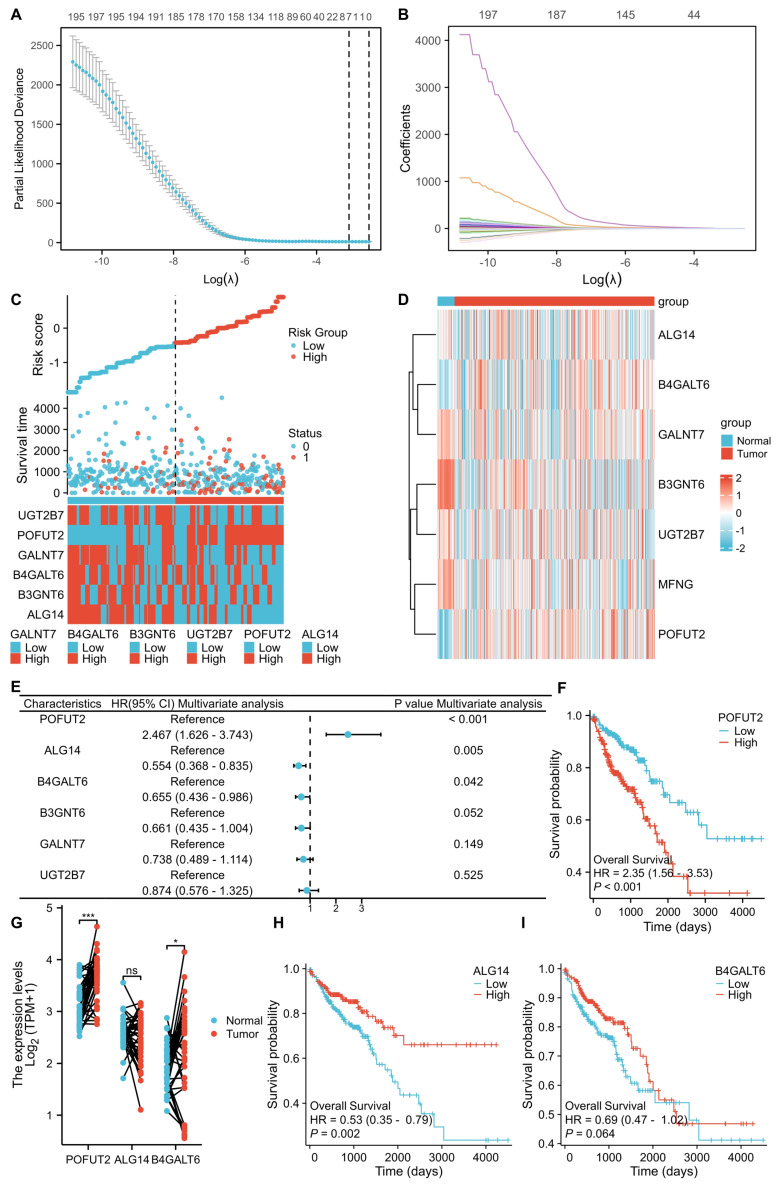
** Identifying key glycosyltransferases in colorectal cancer. (A)** Cross-validation was utilized to optimize parameter selection within proportional hazards models.** (B)** Distribution of LASSO regression coefficients for 200 glycosyltransferase genes. **(C)** Distribution of risk scores, survival status, and expression of risk-associated genes in high- and low-risk groups within the TCGA CRC dataset (n=521 patients).** (D)** Heatmap representation of the expression of glycosyltransferase signature genes in the TCGA CRC dataset. **(E)** Multivariate Cox proportional hazards regression analysis of the core glycosyltransferase genes. **(F,H-I)** Kaplan-Meier survival curves for POFUT2, ALG14, and B4GALT6 expression in the TCGA CRC cohort. **(G)** mRNA expression levels of POFUT2, ALG14, and B4GALT6 in the TCGA CRC dataset. Statistical significance is denoted as *P < 0.05, **P < 0.01, ***P < 0.001, ns: not significant (P > 0.05).

We obtained a comprehensive list of GTs from the HGNC database (https://www.genenames.org/). After excluding pseudogenes, the gene expression profiles and association with prognosis of 200 GTs were analyzed using TCGA CRC database. LASSO regression analysis was performed to determine the optimized gene set, with the parameters illustrated in Figure 1A and B. When the lambda (λ) value was minimized, a prognostic model with seven genes having non-zero coefficients (POFUT2, ALG14, B3GNT6, GALNT7, B4GLT6, UGT2B7, and MFNGEGR3, CCND2, SOCS3, JunD, and SLC27A6) was established. The entire CRC cohort was stratified into high and low-risk groups based on the risk score, revealing a significantly higher mortality rate in the high-risk group compared to the low-risk group (Figure 1C). We further explored the correlation between the expression of these seven key genes and clinicopathological characteristics within the TCGA CRC dataset (Figure S1B). Through univariate and multivariate Cox proportional hazards regression analyses, we found that POFUT2 (P<0.001), ALG14 (P=0.005), and B4GALT6 (P=0.042) were independent risk factors affecting CRC patient prognosis (Figure S1A and Figure 1C). Notably, POFUT2 and B4GALT6 showed higher expression in CRC tissues compared to paired normal tissues, while no difference observed for ALG14 expression (Figure 1D,G). In CRC, the upregulation of POFUT2 (P < 0.001) and the downregulation of ALG14 (P = 0.002) were significantly associated with poor overall survival (OS), as determined by Kaplan-Meier survival analysis. In contrast, the expression of B4GALT6 did not show a correlation with OS (Figure 1F,H-I). Based on these findings, we prioritized POFUT2 for further investigation due to its high expression in CRC tissues and profound influence on poor prognosis in CRC.

### POFUT2 is highly expressed in colorectal cancer and is a pro-angiogenic gene

**Figure 2:**
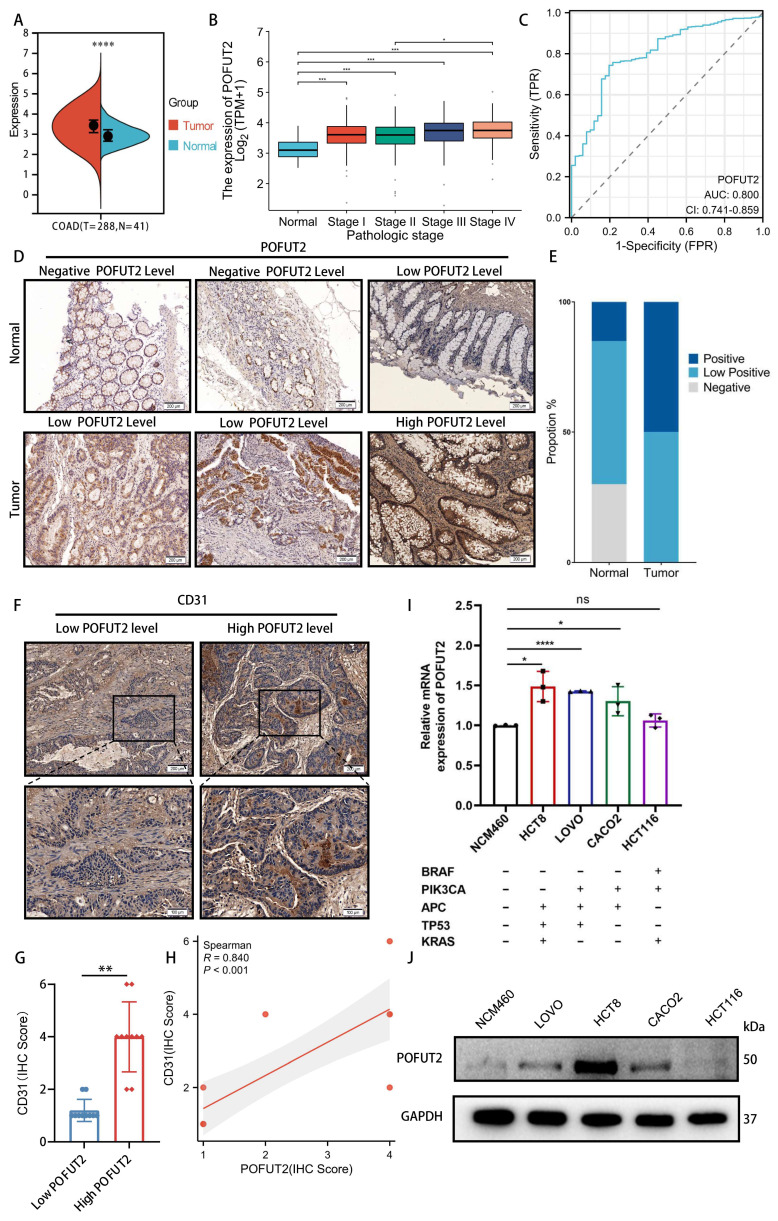
** POFUT2 is highly expressed in colorectal cancer and is a pro-angiogenic gene. (A)** Comparison of POFUT2 expression levels between CRC tissues and their paired adjacent normal samples. **(B)** Variation in POFUT2 expression across different pathological stages CRC. **(C)** ROC curves for POFUT2.** (D-E)** Immunohistochemical detection and statistical scoring of POFUT2 protein expression levels in tumors and paracancerous tissues from 20 CRC patients.** (F)** CD31 staining results in 20 tissue specimens, categorized into high and low POFUT2 expression groups.** (G)** Histogram showing the difference in CD31 expression levels between the high and low POFUT2 expression groups.** (H)** Correlation analysis between POFUT2 staining scores and CD31 scores.** (I)** Quantitative RT-PCR analysis of POFUT2 mRNA expression levels across different CRC cell lines.** (J)** Western blot analysis of POFUT2 protein expression levels in various CRC cell lines. Data are statistically analyzed using a T-test or Mann-Whitney U.*P < 0.05; **P < 0.01; ***P < 0.001; ****p < 0.0001 indicate statistical significance.

POFUT2 expression was found to be elevated in CRC tissues compared to paired normal tissues in TCGA CRC cohort (Figure 2A). This increase in expression was observed in conjunction with higher tumor infiltration depths (T stage), more extensive lymphatic metastasis (N stage), presence of distant metastasis (M stage), and advanced clinicopathological staging (Figure 2B and Figure S1C-E). Additionally, we conducted an analysis of the receiver operating characteristic (ROC) curve for POFUT2 and found it demonstrated a substantial diagnostic value, evidenced by an area under the curve (AUC) of 0.8(Figure 2C). In comparison, the classic CRC marker, carcinoembryonic antigen (CEA/CEACAM5), exhibited a lower AUC of 0.504 (Figure S2A-C). Immunohistochemical analysis of 20 pairs of CRC surgical specimens and adjacent non-cancerous tissues further corroborated that POFUT2 protein expression was significantly elevated in cancerous tissues (Figure 2D-E). Thus, POFUT2 is overexpressed in CRC and influences patient prognosis which maybe as a predictive and prognostic biomarker for CRC outcomes.

To investigate the potential role of POFUT2 in CRC, we first used the CancerSEA database (http://biocc.hrbmu.edu.cn/CancerSEA/) and found a robust positive correlation between POFUT2 expression and the angiogenic phenotype in CRC (Figure S2D). Based on the immunohistochemical staining scores of POFUT2 in 20 colorectal cancer tissues, we categorized the samples into two groups: the high POFUT2 expression group (n=10) and the low POFUT2 expression group (n=10). Immunohistochemical staining of these specimens with a CD31 antibody showed significantly higher CD31 expression in the high POFUT2 level group than in the low-level group, along with a strong positive correlation between CD31 and POFUT2 protein expression (r=0.840, P<0.001) (Figure 2F-H). Next, we evaluated POFUT2 expression in an immortalized colorectal epithelial cell line (NCM460) and several CRC cell lines. Our results indicated that POFUT2 expression was higher in and LOVO, HCT8 and CACO2 CRC cell lines compared to NCM460 cells (Figure 2I-J). Based on these expression levels, we selected the two cell lines with the highest POFUT2 expression, HCT8 and LOVO, for subsequent *in vitro* experiments. Altogether, these findings suggest that POFUT2 may play a promotional role in the angiogenesis of CRC.

### High expression of POFUT2 in CRC cells promotes the angiogenic capacity of HUVECs

**Figure 3:**
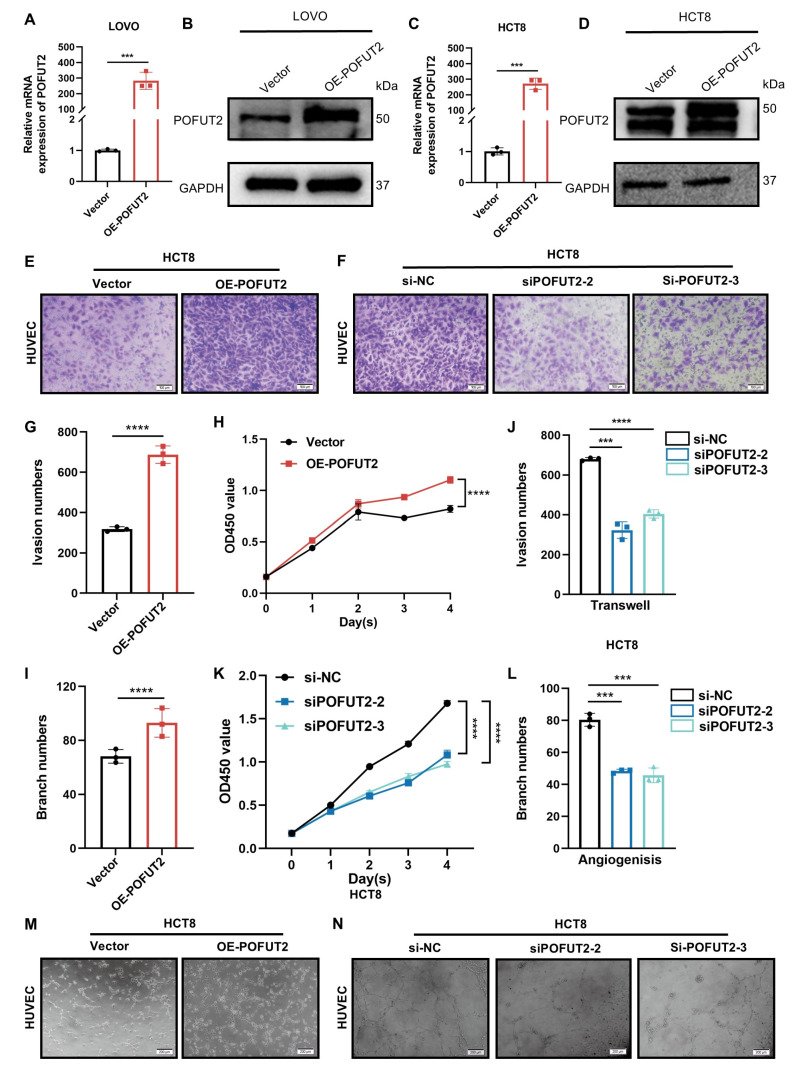
** High expression of POFUT2 in CRC cells promotes the angiogenic capacity of HUVECs. (A, C)** Quantitative RT-PCR (qRT-PCR) was used to assess the overexpression efficiency of POFUT2 in HCT8 and LOVO cells.** (B, D)** Western blot analysis was conducted to evaluate the overexpression efficiency of POFUT2 in HCT8 and LOVO cells.** (E-F,G,J)** Transwell invasion assays, along with quantitative results, were conducted on HUVECs cultured with TCM from HCT8 cells overexpressing or knocking down POFUT2.** (H,K)** CCK-8 proliferation assays were conducted on HUVECs cultured with TCM derived from HCT8 cells that had been transfected with either a POFUT2 overexpression plasmid or siRNA targeting POFUT2. **(I,L,M-N)** Tubulogenesis assays, along with quantitative results, were conducted on HUVECs cultured with TCM from HCT8 cells overexpressing or knocking down POFUT2. Data are presented as mean ± standard deviation, with statistical analysis performed using a T-test, n = 3, ***P < 0.001; ****p < 0.0001 indicating statistical significance.

To elucidate the role of POFUT2 in the regulation of angiogenesis in CRC, we transfected HCT8 and LOVO cells with a POFUT2 overexpression plasmid. This transfection successfully increased POFUT2 expression at both the mRNA and protein levels in these cells (Figure 3A-D). Subsequently, we initially confirmed the efficiency of POFUT2 knockdown in HCT8 and LOVO cells, demonstrating that POFUT2 expression was significantly reduced compared to negative controls (Figure S2E-H). Our results showed that HUVECs cultured in TCM from POFUT2-overexpressing HCT8 and LOVO cells displayed significantly higher cell proliferation, enhanced angiogenesis, and increased invasion abilities compared to those cultured in TCM from control cells (Figure 3E,G-I,M and Figure S3A,C-E,I). Compared with the TCM derived from the negative control HCT8 cells, the proliferation, angiogenic, and invasion capabilities of HUVECs cultured with TCM collected after knockdown of POFUT2 were significantly reduced (Figure 3F,J-L,N and Figure S3B,F-H,J). Collectively, these findings indicated that POFUT2 promotes the proliferation, angiogenesis, and invasion abilities of HUVECs *in vitro*, highlighting its potential role in the angiogenic processes associated with CRC.

### POFUT2 promotes angiogenesis by upregulating JUP expression

**Figure 4:**
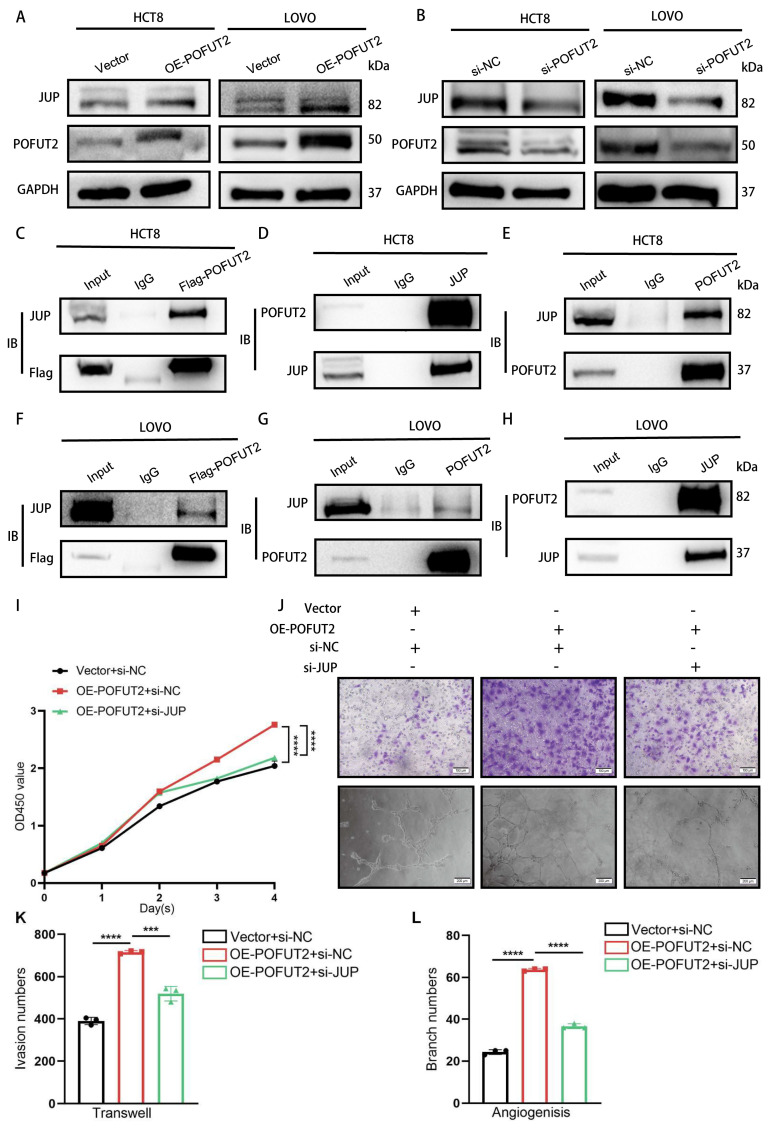
** POFUT2 promotes angiogenesis by upregulating JUP expression. (A-B)** Detection of JUP expression levels by Western Blot after POFUT2 overexpression and knockdown in HCT8 and LOVO cells. **(C,F)** Immunoprecipitation (IP) using an exogenous Flag antibody to pull down POFUT2-bound complexes after POFUT2 overexpression in HCT8 and LOVO cells, with Western Blot detection of JUP expression.**(D,G)** IP experiments using a JUP antibody in HCT8 and LOVO cells, followed by Western Blot detection of POFUT2 expression.**(E,H)** IP experiments using a POFUT2 antibody in HCT8 and LOVO cells, followed by Western Blot detection of JUP expression.** (I-L)** Assessment of TCM from HCT8 cells post-transfection with POFUT2 overexpression plasmid and JUP siRNA on HUVECs for CCK-8 proliferation assays, Transwell invasion, and tubulogenesis, along with quantitative results. Data are presented as mean ± standard deviation, with n = 3, **P < 0.01, ***P < 0.001, ****p < 0.0001 indicating statistical significance as determined by T-test analysis.

To investigate the mechanisms underlying POFUT2-mediated angiogenesis in colorectal cancer, we transfected HCT8 cells with a 3xFLAG-POFUT2 plasmid and employed a FLAG-tag antibody to immunoprecipitate POFUT2-interacting proteins for subsequent mass spectrometry analysis (Figure S4B). There were three potential interacting proteins associated with angiogenesis: JUP, HSPB1, and AGO2.HSPB1 negatively regulates angiogenesis, while JUP and AGO2 positively regulate angiogenesis (Figure S4A). In the TCGA CRC dataset, we observed JUP and AGO2 exhibited upregulated expression in tumor tissues compared with normal tissues (Figure S4C-D). Importantly, JUP protein expression but not mRNA levels increased with POFUT2 overexpression and decreased with POFUT2 knockdown in HCT8 and LOVO cells, respectively (Figure 4A-B and Figure S4F-I). Concurrently, the protein level of AGO did not alter after POFUT2 overexpression (Figure S4J). Based on these findings, we hypothesize that POFUT2 may exert its influence on CRC angiogenesis through enhancing JUP protein level dependent on post-transcriptional regulatory mechanisms.

A pattern also observed in CRC cell lines, which exhibited higher JUP expression than normal colorectal epithelial cell lines (Figure S4K). Immunoprecipitation experiments post-transfection of Flag-POFUT2 in HCT8 and LOVO cells observed the binding of POFUT2 to JUP (Figure 4C and Figure 4F). Endogenous reciprocal IP assays further validated the POFUT2-JUP interaction in CRC cells (Figure 4D-E and Figure 4G-H).

To ascertain whether POFUT2 may promote angiogenesis in CRC by enhancing JUP expression, we overexpressed POFUT2 and simultaneously knocked down JUP in the HCT8 cell line. Subsequently, we collected TCM from these cells and used it to culture HUVECs for Transwell invasion assays, tubulogenesis, and CCK-8 proliferation assays. Our findings indicate that the suppression of JUP expression counteracts the enhanced proliferation, invasiveness, and angiogenic capabilities of HUVECs induced by POFUT2 overexpression (Figure 4I-L). Our findings reveal that POFUT2 promotes angiogenesis in CRC by elevating JUP expression through direct binding to JUP.

### POFUT2 mediated fucosylation of JUP to enhance VEGFA expression

**Figure 5:**
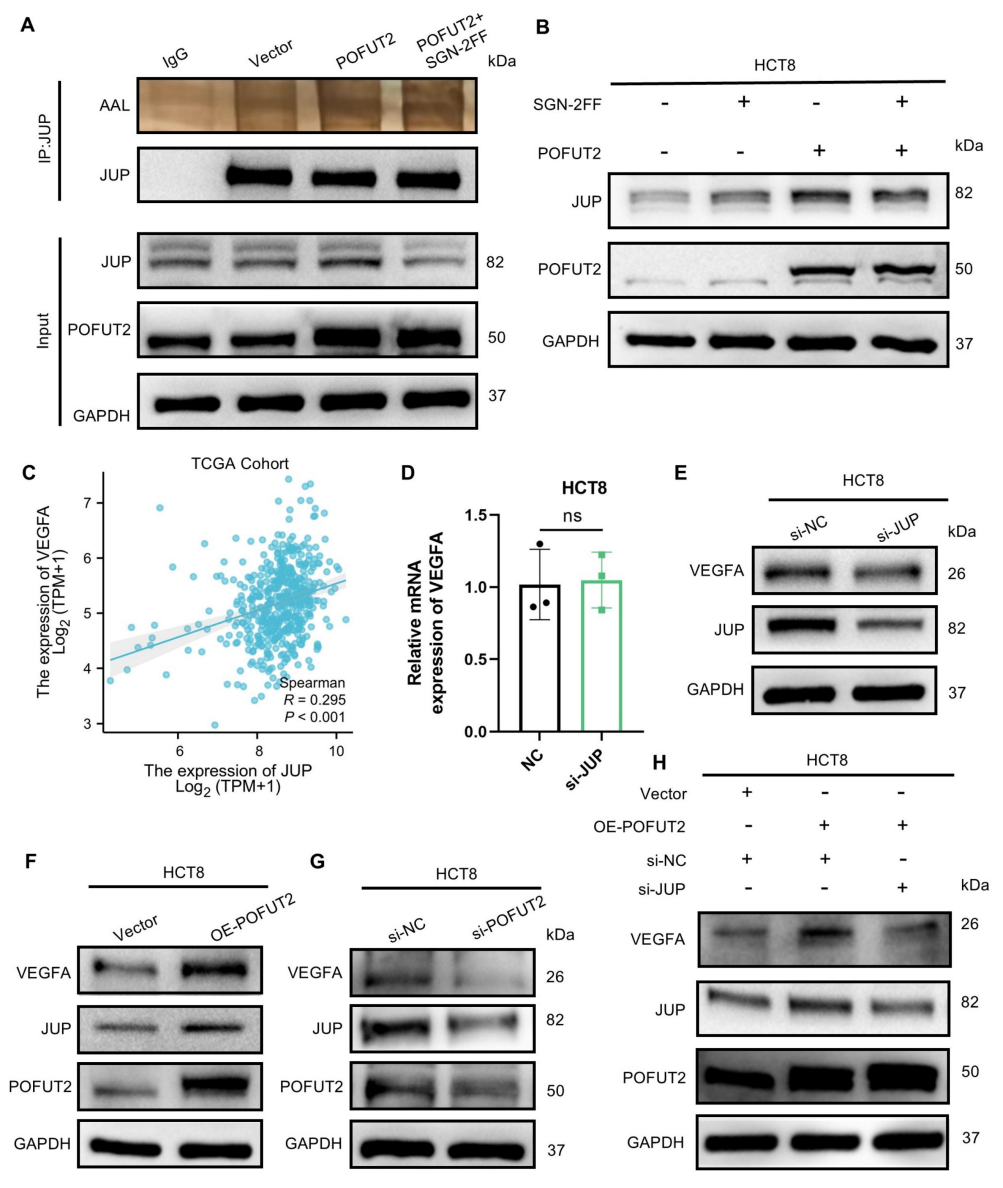
** POFUT2 mediated fucosylation of JUP to enhance VEGFA expression. (A)** Detection of JUP fucosylation by Western Blot after POFUT2 overexpression in HCT8 cells, using an exogenous Flag antibody to pull down POFUT2, followed by lectin AAL blotting. **(B)** Detection of JUP expression level after POFUT2 overexpression in HCT8 cells with a 24-hour treatment of SGN-2FF (10μm). **(C)** Scatterplot illustrating the expression correlation between JUP and VEGFA within the TCGA CRC cohort. **(D)** qRT-PCR detection of VEGFA expression following JUP knockdown in HCT8 cells. **(E)** Western blot analysis of VEGFA protein expression levels after transfection with JUP siRNA in HCT8 cells. **(F-H)** Western blot analysis of VEGFA protein expression levels after transfection with POFUT2 overexpression plasmid and POFUT2 siRNA in HCT8 cells. Data are presented as mean ± standard deviation and were analyzed using T-test statistical analysis, with n = 3, **P < 0.01, ***P < 0.001, ns: not significant (P > 0.05).

Given that POFUT2 is a protein O-fucosyltransferase that adds O-fucose groups to serine/threonine residues, we investigated whether POFUT2 directly modulates the O-fucosylation level of JUP, thereby affecting JUP protein expression. Through immunoprecipitation assay followed by AAL lectin blotting, we found increased AAL binding to JUP in POFUT2-overexpressing cells. This phenomenom was reduced by fucosyltransferase inhibitor (SGN-2FF) (Figure 5A). Overexpression of POFUT2 in HCT8 and LOVO cells resulted in increased JUP protein levels with POFUT2 overexpression, which were also abrogated by SGN-2FF treatment (Figure 5B and Figure S5A). Our findings suggest that POFUT2 interacts with JUP, facilitating its fucosylation modification, which in turn enhances the expression levels of the JUP protein.

JUP is a key player in cellular adhesion and tissue integrity. Overexpression of JUP in melanoma has been shown to upregulate VEGFA expression, and tumors with elevated JUP display a larger area of CD31-positive staining, indicating increased angiogenesis^35^. In the TCGA CRC dataset, a significant positive correlation was observed between JUP and VEGFA expression levels (R = 0.295, P < 0.001) (Figure 5C). VEGFA is recognized as one of the most potent angiogenic factors, and the VEGFA/VEGFR pathway plays a crucial role in promoting the development of vascular endothelial cells during tumor angiogenesis^36^. We hypothesize that POFUT2 may promote angiogenesis in CRC by regulating VEGFA through JUP. In the TCGA CRC dataset, a positive correlation was observed between JUP and VEGFA expression levels (R = 0.295, P < 0.001) (Figure 5C). Our data reveal that knocking down JUP in CRC cells leads to a decrease in VEGFA protein levels without affecting VEGFA mRNA levels (Figure 5D-E and Figure S5D-E). Similarly, we observed VEGFA protein increased upon POFUT2 overexpression and decreased following POFUT2 knockdown in HCT8 cells (Figure 5F-G and Figure S5F-I). When POFUT2 was overexpressed concurrently with JUP knockdown, the increased VEGFA expression due to POFUT2 overexpression was counteracted, returning to basal levels (Figure 5H). Collectively, these findings suggest that POFUT2 promotes angiogenesis in CRC by increasing VEGFA expression through the direct regulation of O-fucosylation modification of JUP.

### Significant positive correlation between POFUT2 expression and JUP/VEGFA expression in colorectal cancer tissues

**Figure 6:**
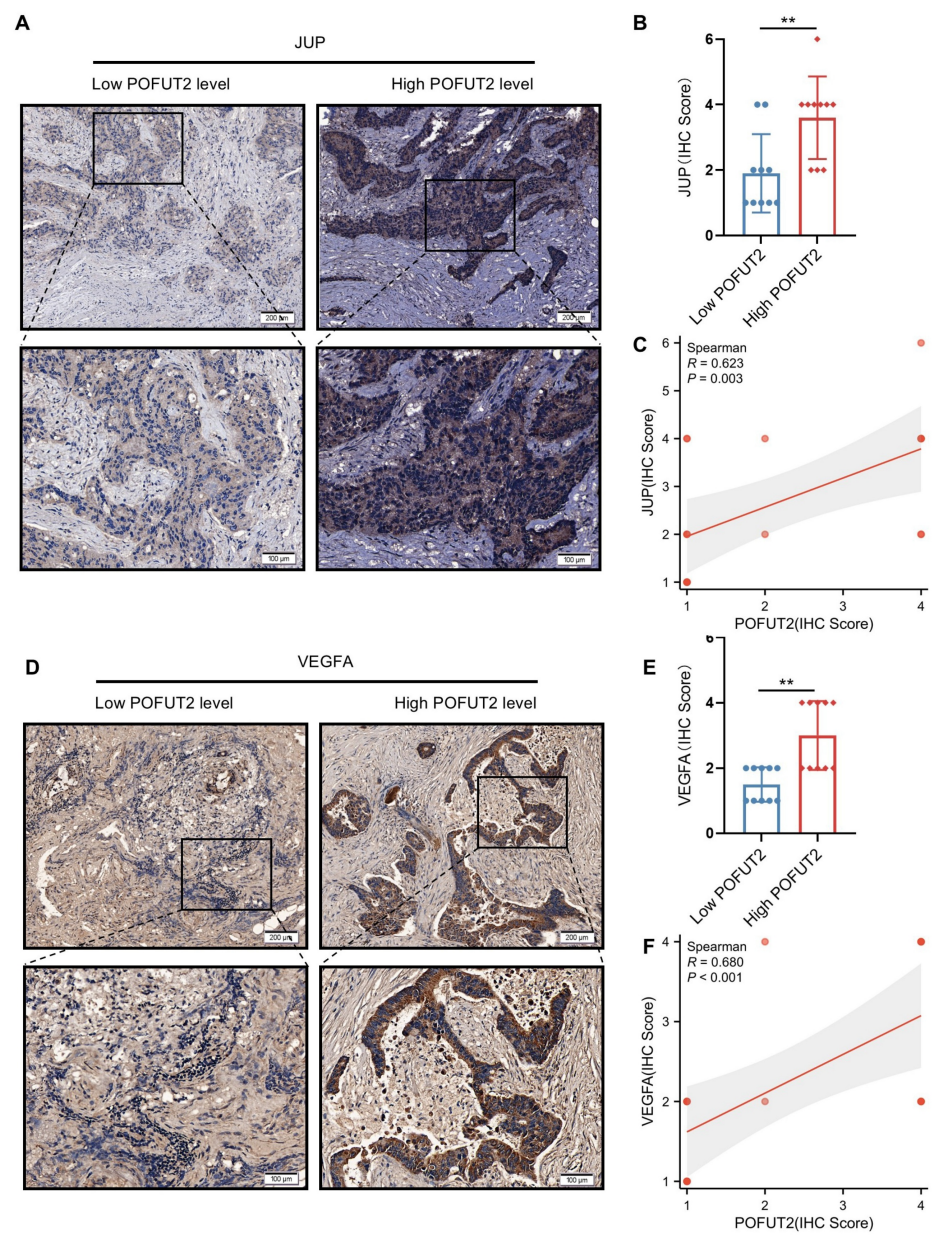
** Significant positive correlation between POFUT2 expression and JUP/VEGFA Expression in Colorectal Cancer Tissues. (A, D)** Staining results for JUP/VEGFA after stratifying 20 tissue samples into high and low POFUT2 expression groups. **(B, E)** Histograms illustrating the differences in JUP/VEGFA expression between the high and low POFUT2 expression groups. **(C, F)** Correlation between POFUT2 staining scores and CD31 scores. Data are presented as mean ± standard deviation, and statistical significance was determined using a Mann-Whitney U. *P < 0.05, **P < 0.01, ***P < 0.001 ns: not significant (P > 0.05).

Subsequently, we aimed to investigate whether a similarly robust positive correlation exists between POFUT2 and JUP/VEGFA in CRC cancerous tissues. We stratified 20 CRC tissues into groups with high and low POFUT2 expression and further examined the expression patterns of JUP and VEGFA within these groups. Immunohistochemical staining revealed that, compared to the low POFUT2 level group, the high POFUT2 level group exhibited significantly elevated protein levels of JUP and VEGFA (Figure 6A-B, D-E). Notably, a significant positive correlation was observed between POFUT2 and the protein expression levels of JUP and VEGFA in CRC tissues (JUP: r=0.623, p=0.003; VEGFA: r=0.680, p<0.001) (Figure 6C, F). These findings further corroborate our hypothesis that POFUT2 promotes angiogenesis in CRC through the JUP/VEGFA axis.

## Discussion

In our study, we identified POFUT2 as a potential oncogene glycosyltransferase that is highly expressed at multiple levels—TCGA dataset, CRC cell lines, and CRC tissues—and serves as an independent risk factor impacting the prognosis of CRC patients. ROC curve revealed that POFUT2 has a significantly higher AUC value compared to the established CRC markers CEA/CEACAM5, underscoring the substantial potential of POFUT2 as a molecular diagnostic biomarker for CRC. Utilizing the cancerSEA database, we identified a strong correlation between POFUT2 expression and the angiogenic phenotype, suggesting a potential role in vascular biology. This hypothesis was further supported by our immunohistochemical staining in CRC tissues, which revealed a significant positive correlation between POFUT2 and the expression of the vascular marker CD31. Our findings demonstrate that POFUT2 knockdown in CRC cells inhibits the proliferation, invasion, and angiogenic capacity of HUVECs, while POFUT2 overexpression significantly promotes ability of HUVECs.

Through mass spectrometry detection, we identified POFUT2 binding protein JUP. We found POFUT2 directly interacted with and modulated its protein expression levels by engaging in the fucosylation modification of JUP. JUP is known to interact with axonemal calreticulin, axonemal core glycoproteins, and other axonemal proteins, which facilitate the binding of intermediate filaments to the cell membrane, thereby conferring cellular tensile strength and elasticity. JUP also binds to APC, which, in conjunction with axin and GSK3, regulates JUP levels within the cytoplasm and nucleus 37,38. Activation of the Wnt pathway leads to the accumulation of JUP in the cytoplasm and nucleus, where it binds to members of the Tcf/Lef family of transcription factors, increasing the expression of cell cycle proteins D1, c-myc, and MMP-7^18^. Moreover, JUP overexpression significantly enhances the expression of VEGFA^35^. Our experimental results indicate that POFUT2 and JUP do not influence the mRNA expression levels of VEGFA but rather modulate its protein expression. We observed that when POFUT2 was overexpressed and JUP was concurrently knocked down, the increased VEGFA protein expression induced by POFUT2 overexpression returned to baseline levels. This discrepancy suggests that POFUT2-JUP axis regulation of VEGFA expression may mainly occur at the post-transcriptional level, rather than at the transcriptional level. Moreover, the knockdown of JUP reversed the enhancement of proliferation, invasion, and angiogenic capabilities of HUVECs induced by POFUT2 overexpression. Further analysis in CRC tissues revealed a significant positive correlation between POFUT2 expression and the protein expression levels of JUP and VEGFA.

The standard first-line treatment for advanced CRC remains chemotherapy in combination with anti-epidermal growth factor receptor (EGFR) antibodies or anti- VEGF agents^5^,^39^-^41^. While the clinical benefits of anti-angiogenic therapies such as bevacizumab are substantial, they have not matched the high expectations set by preclinical research^42^-^44^. This discrepancy is largely attributed to the significant side effects and the development of drug resistance^45^. Consequently, there is an urgent need to discover new drug targets based on underexplored mechanisms to combat tumor angiogenesis. Recent studies have identified four potential POFUT2-targeting drugs with plausible docking modes that may be effective against chemotherapy-resistant cancers^46^. In our future work, we plan to investigate the potential synergistic effects of POFUT2 inhibitors in combination with bevacizumab for the treatment of CRC.

While our study has elucidated the molecular mechanism by which POFUT2 promotes angiogenesis in colorectal cancer through the regulation of JUP and VEGFA, several limitations should be acknowledged. The sample size in our study is relatively small. Larger patient cohort studies are warranted to further validate the role of POFUT2 across different clinical subtypes and its correlation with patient prognosis. In addition, although we identified the interaction between POFUT2 and JUP using co-immunoprecipitation in cells, the specificity binding domain of this interaction requires further confirmation through additional independent experiments. Moreover, while *in vitro* functional assays have provided compelling evidence for the function of POFUT2, the *in vivo* validation of these experimental results remains to be fully explored. In our future work, we plan to conduct intestinal-specific POFUT2 knockout mice to further investigate the function and mechanisms of POFUT2 on CRC progression.

**Figure 7:**
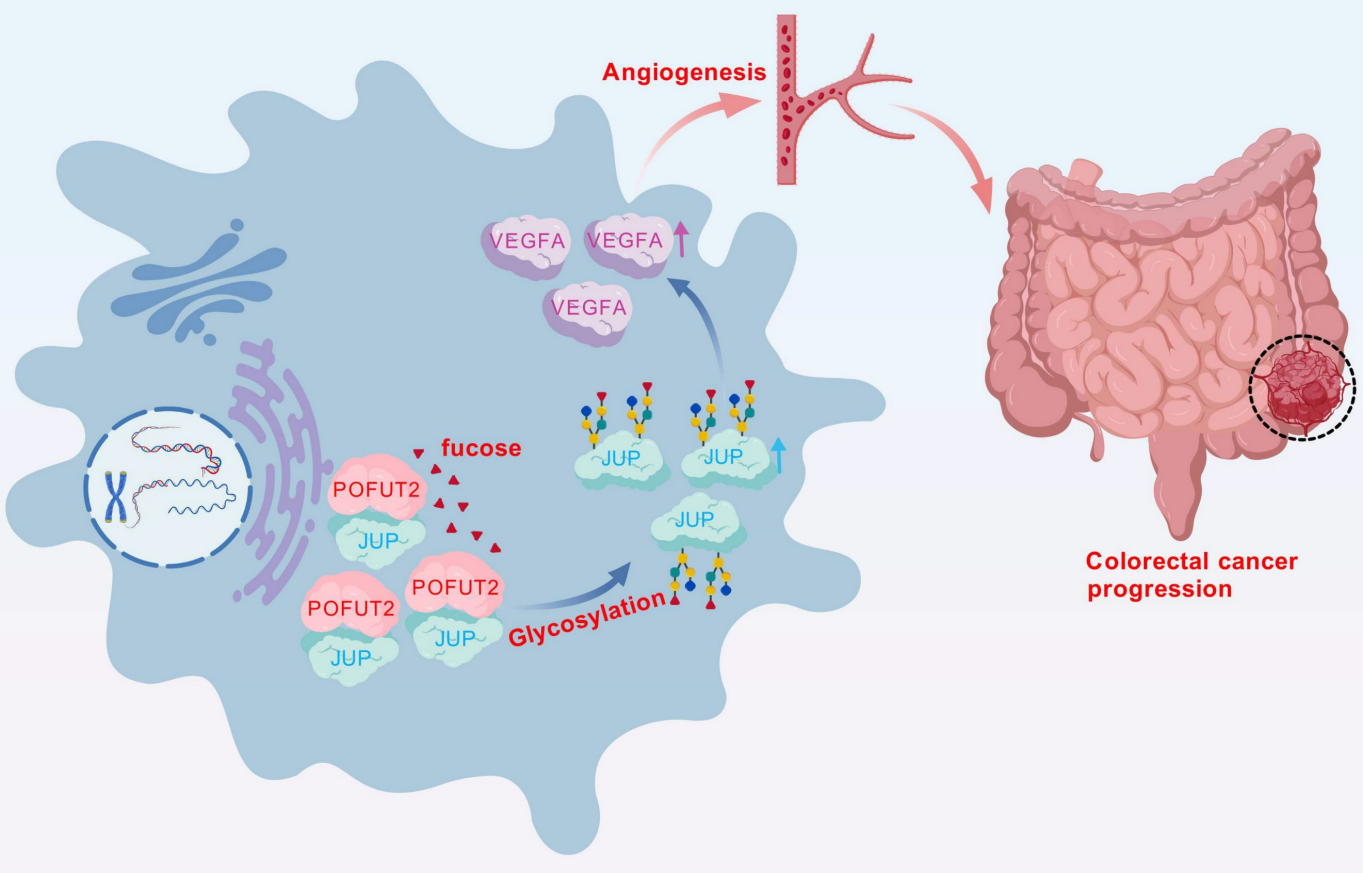
In CRC, POFUT2 engages in an interaction with JUP that results in the fucosylation of JUP, which in turn elevates the protein expression levels of JUP. Subsequently, POFUT2, through its influence on JUP, stimulates the expression of VEGFA, thereby promoting the angiogenic process in CRC.

In conclusion, our study of key glycosyltransferases has uncovered a novel mechanism of angiogenesis in CRC. We have demonstrated that high expression levels of POFUT2 in CRC are implicated in the regulation of fucosylation modification levels of JUP, which subsequently increases JUP protein expression. Furthermore, POFUT2 exerts its effects on VEGFA protein expression through JUP, thereby promoting angiogenesis in CRC (Fig. 7). These findings not only advance our understanding of the molecular underpinnings of CRC angiogenesis but also suggest POFUT2 as a potential anti-angiogenic therapeutic target. Targeting POFUT2 could have significant implications for the clinical management of CRC, offering a new avenue for treatment strategies in patients with this disease.

## Supplementary Material

Supplementary methods and figures.

## Abbreviations

CRC: colorectal cancer
TCGA: the cancer genome atlas
POFUT2: GDP-fucose protein o-fucosyltransferase 2
HUVECs: human umbilical vein endothelial cells
JUP: junction plakoglobin
VEGFA: vascular endothelial growth factor A
CD31: platelet endothelial cell adhesion molecule-1
TCM: tumor conditioned medium
OS: overall survival
SGN-2FF: fucosyltransferase inhibitor
