# Supplementary material for: POFUT2 Mediated Fucosylation of JUP Enhances VEGFA Expression to Promote Angiogenesis in Colorectal Cancer
Source: Int J Med Sci. 2026 Jan 1;23(2):412–27. doi: 10.7150/ijms.113515 (PMC12825011; doi:10.7150/ijms.113515)
Supplement: Supplementary file 1 — Supplementary methods and figures. [file ijmsv23p0412s1.pdf]

POFUT2 Mediated Fucosylation of JUP Enhances VEGFA Expression to Promote Angiogenesis in Colorectal Cancer

Yanfeng Zou[1#],Yian Yang[1],Wei Xu[2],Peiguo Cao[1*],Zheng Li[2*]

[1]Department of Oncology, Third Xiangya Hospital, Central South University, Changsha 410013, China.

[2] NHC Key Laboratory of Carcinogenesis, Key Laboratory of Carcinogenesis, Chinese Ministry of Health, Cancer Research Institute, Xiangya School of Basic Medical Sciences, Central South University, Changsha, Hunan, China.

*Correspondence

Dr. Peiguo Cao

E-mail: xy3caopg@csu.edu.cn

Dr. Zheng Li

E-mail: lizheng@csu.edu.cn

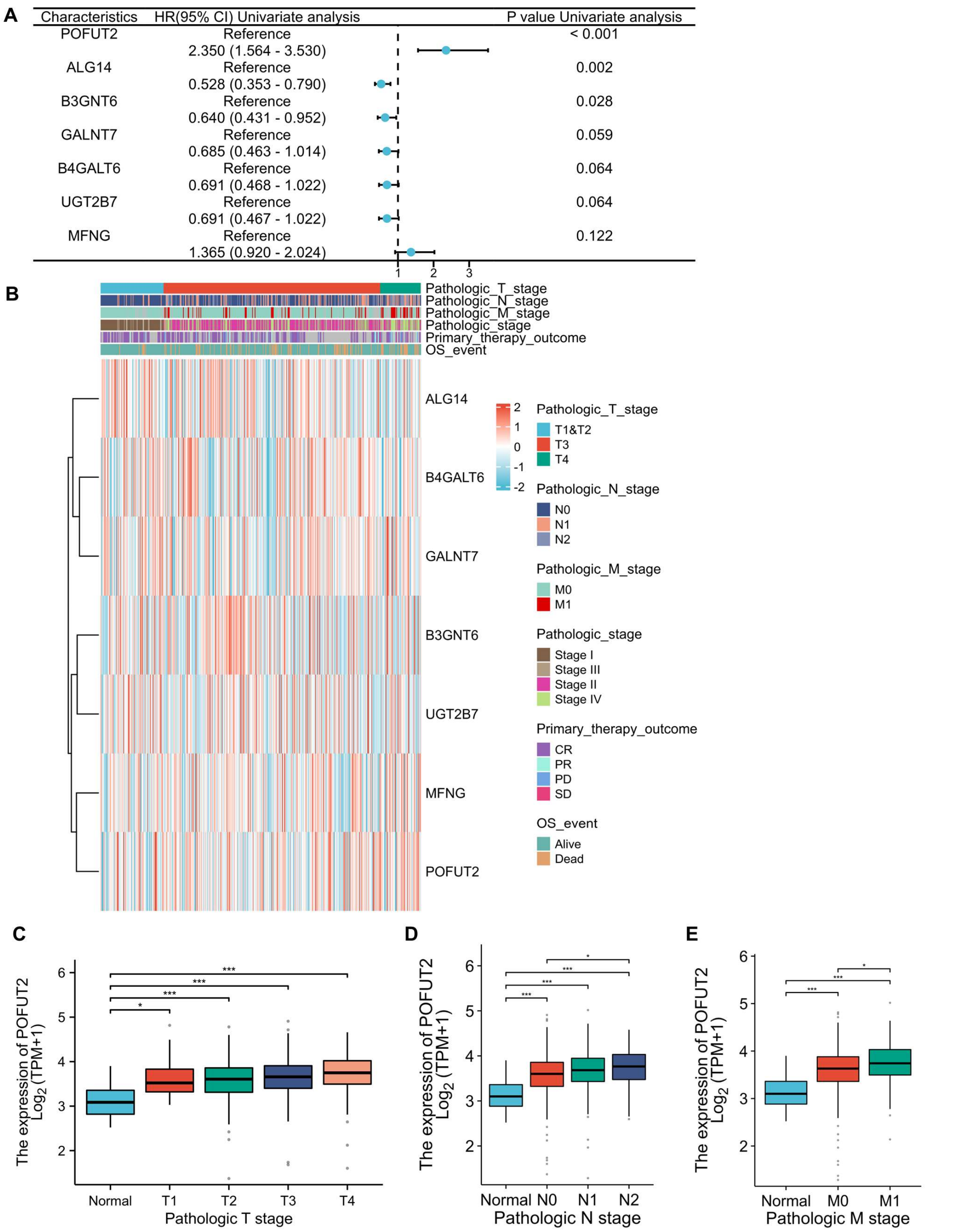

Figure S1

Identifying key glycosyltransferases in colorectal cancer.(A) Univariate Cox proportional hazards regression analysis for the core glycosyltransferase genes.**(B)**Heatmap illustrating the correlations between the differential expression of seven glycosyltransferase genes and various clinicopathological features. **(C)**Variation in POFUT2 expression across different T-stages of

CRC.**(D)**Variation in POFUT2 expression across different N-stages of CRC.**(E)** Variation in POFUT2 expression across different M-stages of CRC. *P < 0.05, **P < 0.01, ***P < 0.001 ns: not significant (P > 0.05).

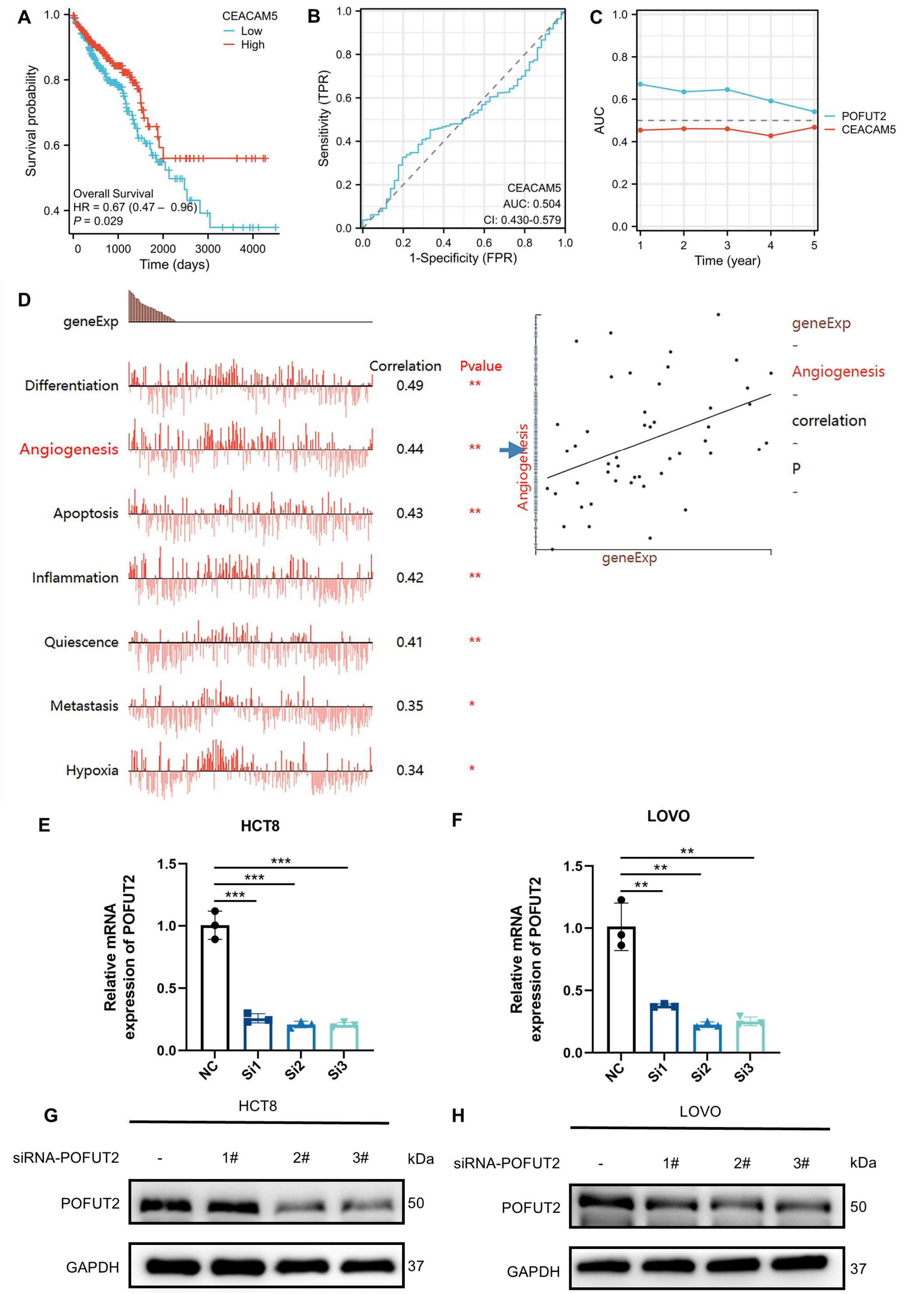

Figure S2

POFUT2 is highly expressed in colorectal cancer and is a pro-angiogenic gene.(A) Kaplan-Meier survival curves for CEA/CEACAM5 in the TCGA CRC cohort. **(B)** Receiver operating characteristic (ROC) curves for CEA/CEACAM5.**(C)** Time-dependent area under the curve (AUC) comparison of POFUT2 versus CEA/CEACAM5. **(D)** Scatterplot illustrating the correlation between POFUT2 expression and the predicted angiogenic phenotype as analyzed by the CancerSEA database. **(E-F)** The knockdown efficiency of POFUT2 in HCT8 and LOVO cells was assessed using qRT-PCR following transfection with siRNA. **(G-H)** Protein knockdown efficiency of POFUT2 in HCT8 and LOVO cells was evaluated by Western blot after siRNA transfection.Data are statistically analyzed using a T-test and presented as mean ± standard deviation, n = 3, ***P < 0.001 indicating statistical significance.

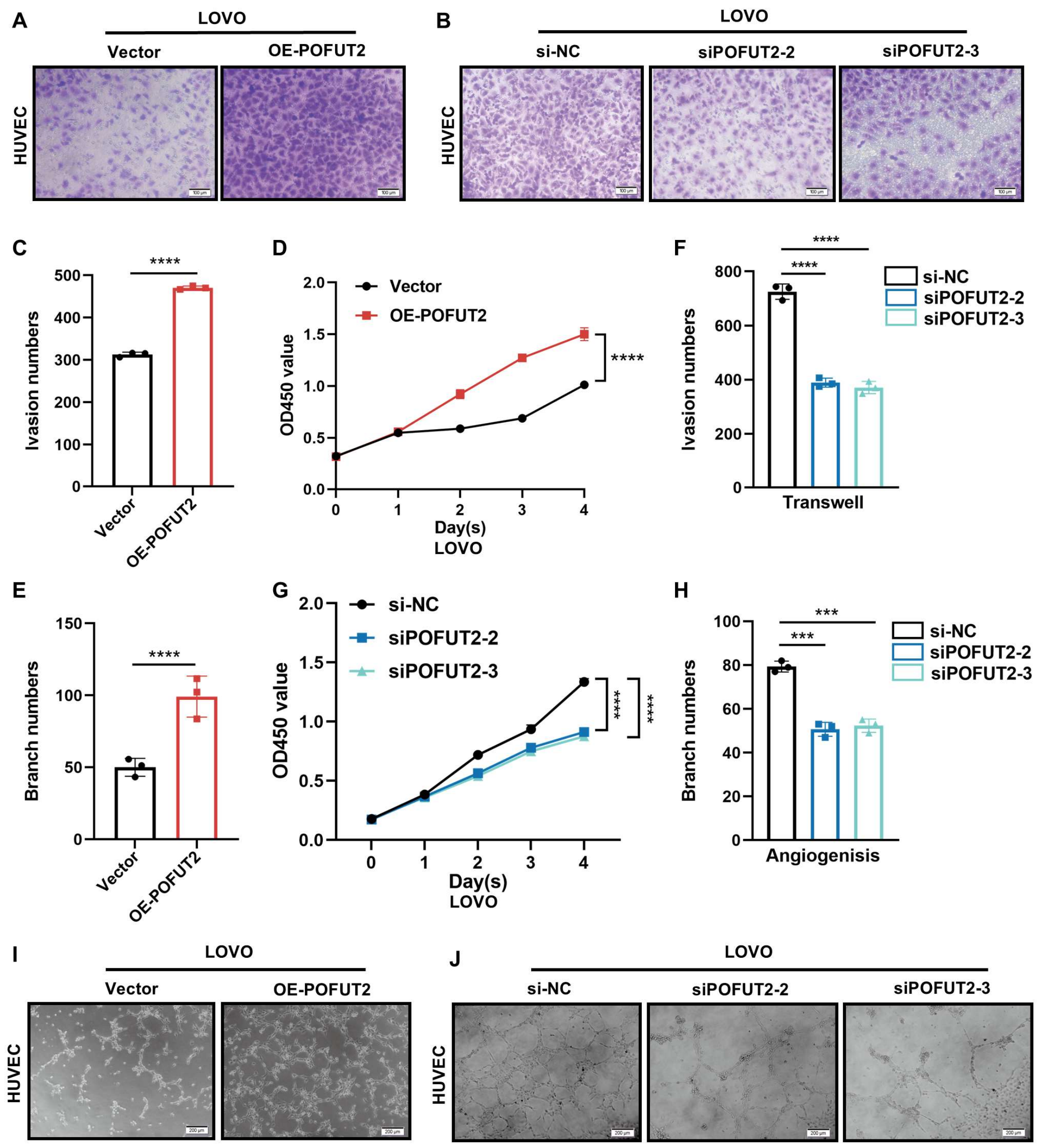

Figure S3

High expression of POFUT2 in CRC cells promotes the angiogenic capacity of HUVECs.(A-B,C,F) Transwell invasion assays, along with quantitative results, were conducted on HUVECs cultured with TCM from LOVO cells overexpressing or knocking down POFUT2. **(D,G)** CCK-8 proliferation assays were conducted on HUVECs cultured with TCM derived from LOVO cells that had been transfected with either a POFUT2 overexpression plasmid or siRNA targeting POFUT2. **(E,H,I-J)** Tubulogenesis assays, along with quantitative results, were conducted on HUVECs cultured with TCM from LOVO

cells overexpressing or knocking down POFUT2. Data are presented as mean ± standard deviation, with n = 3, **P < 0.01, ***P < 0.001,****p < 0.0001 indicating statistical significance as determined by T-test analysis.

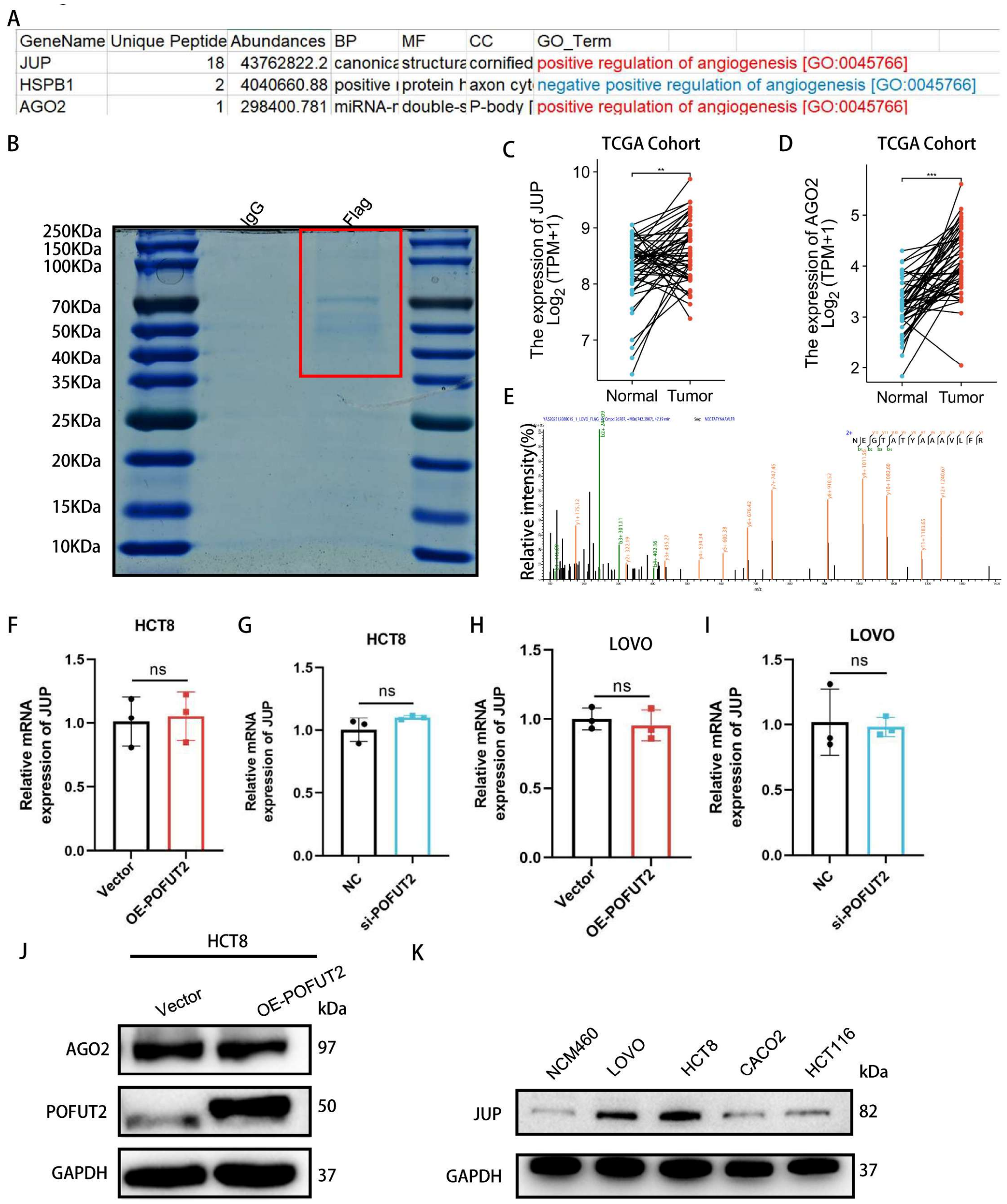

Figure S4

POFUT2 promotes angiogenesis by upreglating JUP expression. (A) Gene Ontology (GO) enrichment analysis for three genes associated with

angiogenesis.**(B)** Immunoprecipitation (IP) experiments were conducted using an exogenous Flag antibody in HCT8 cells after transfection with a POFUT2 overexpression plasmid, with positive bands identified after staining with Coomassie Brilliant Blue. **(C)** Plot showing differential JUP expression in the TCGA CRC cohort. **(D)** Plots depicting the differential expression of AGO2 in the TCGA CRC cohort. **(E)** Mass spectrometry analysis of JUP protein interactions. **(F-I)** Detection of JUP expression by qRT-PCR after POFUT2 overexpression and knockdown in HCT8 and LOVO cells. **(J)** After overexpressing POFUT2 in HCT8 cells, we employed Western Blot analysis to assess the protein expression levels of AGO2. **(K)** Western blot analysis of JUP protein expression levels across different CRC cell lines.

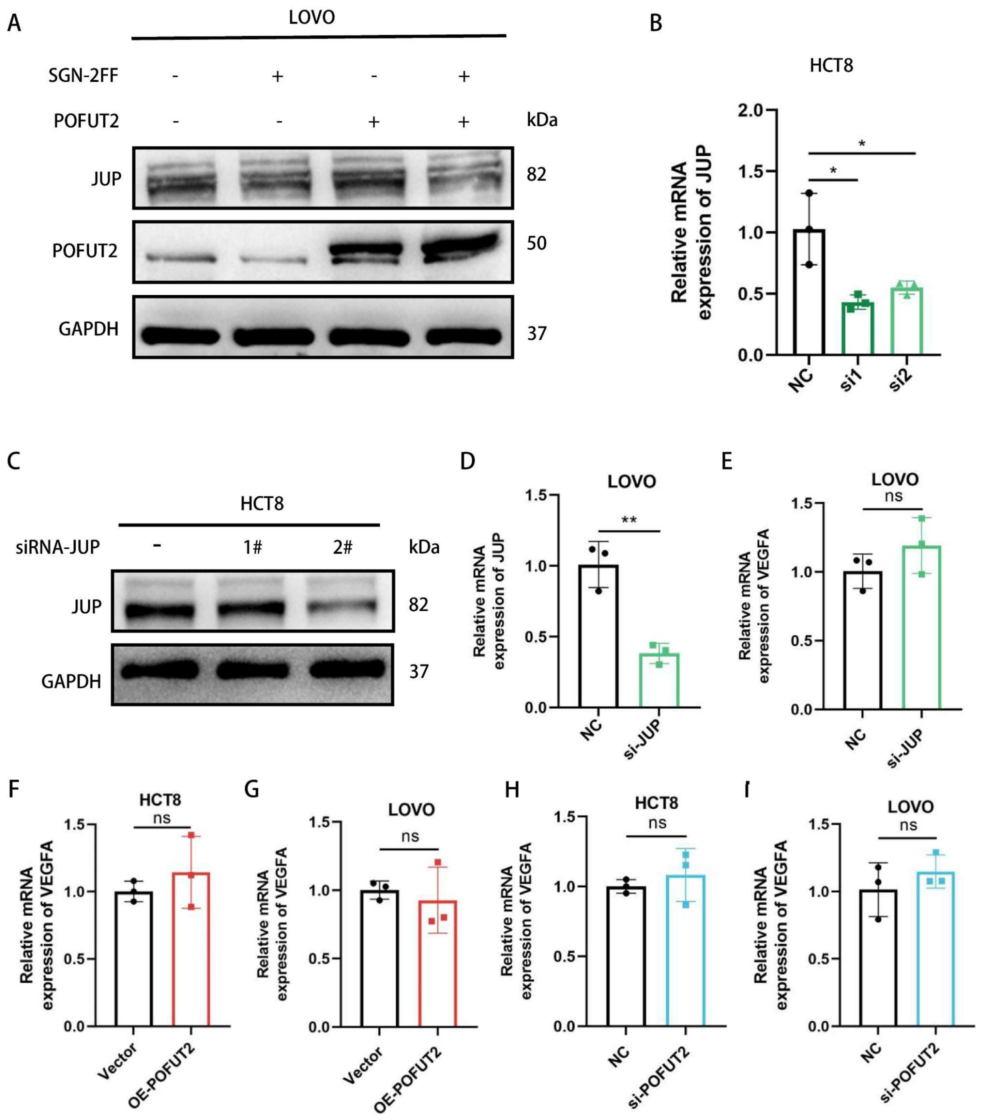

Figure S5

POFUT2 Mediated Fucosylation of JUP to Enhance VEGFA Expression. (A) Detection of JUP expression level after POFUT2 overexpression in LOVO cells with a 24-hour treatment of SGN-2FF. **(B-C)** Knockdown efficiency of JUP detected by qRT-PCR and Western Blot assay following transfection of siRNA in HCT8 cells. **(D-E)**qRT-PCR detection of VEGFA expression following JUP knockdown in LOVO cells. **(F-I)** qRT-PCR detection of VEGFA expression following POFUT2 overexpression and knockdown in HCT8 and LOVO cells. Data are presented as mean ± standard deviation and were analyzed using T-test statistical analysis, with n = 3, $**P < 0.01$, $***P < 0.001$ indicating statistical

significance.

Materials and Methods

Patients and samples

The study involved 20 pairs of tissue samples and corresponding patient data, which were procured from Central South University. Ethical approval for this research was granted by the Ethical Review Committee of the Third Xiangya Hospital, affiliated with Central South University. Prior to their participation, all patients provided their written informed consent, ensuring compliance with ethical standards and patient autonomy.

Cell lines and cell culture

We obtained the human immortalized colorectal epithelial cell line NCM460, as well as human CRC cell lines HCT8, LOVO, CACO2, HCT116, and HUVECs from the Cell Center of Central South University. All cell lines were cultured in Dulbecco's Modified Eagle Medium (DMEM) supplemented with 10% fetal bovine serum. The cells were maintained in a controlled environment of a 37℃ incubator with a 5% CO2 atmosphere to ensure optimal growth conditions.

Cells transfection and tumor conditioned medium (TCM) obtaining

Colorectal cancer cells were transfected with either a pcDNA3.1-3xFlag-POFUT2 overexpression plasmid or siRNA targeting POFUT2. For controls, we used an empty plasmid in the overexpression group and si-NC (non-targeting control siRNA) in the knockdown group. HCT8 and Lovo cells were transiently transfected using the jetPRIME transfection reagent (jetPRIME, polyplus, 101000001). Twelve hours post-transfection, the culture medium was replaced with serum-free medium.TCM were collected 36 hours later. HUVECs were

then cultured in the presence of TCM and subjected to a series of functional assays, including CCK-8 proliferation assay, tube formation assay, and Transwell invasion assay. The specific siRNA sequences used were as follows: for POFUT2, (1:F5′-GGAUGAAGAUGAAGGUCAAT-3′;2: F5′-GGAUGUACCCAGUCUGGAAT-3′; 3:F 5′- CGACCACUAUGGAGGGAAAT-3′), for JUP, (1:F5′-GCTTCAGACTCAAGTACCCA-3′;2: F5′-GATCATGCGTAACTACAGTTA -3′)

CCK-8 proliferation assay

HUVECs were first digested and then seeded into 96-well plates at a density of 1000 cells per well. The culture medium was mixed with TCM in a 1:1 ratio, with a total volume of 100 μL per well. Following this, 10 μL of the Cell Counting Kit-8 (CCK-8, NCM Biotech, C6005) was added to each well. The plates were then returned to the incubator for an additional 2 hours to allow for colorimetric reaction. Subsequently, the absorbance at 450 nm was determined using a microplate reader to assess cell viability.

Tubule formation assay

We utilized pure Matrigel (Corning, 356234) to coat a 24-well plate. The Matrigel was allowed to polymerize for 3 hours at a constant temperature of 37°C in an incubator. Subsequently, HUVECs were seeded onto the Matrigel-coated wells at a density of 8 × 10^4 cells per well. The culture medium was then mixed with TCM in a 1:1 ratio, resulting in a final volume of 400 μL per well. The 24-well plate was incubated for an additional 6 hours in a thermostatic incubator. Post-incubation, the cells were examined under a microscope to evaluate their behavior and morphology within the Matrigel matrix.

Transwell invasion experiment

The substrate gel was diluted with culture medium at a ratio of 1:8. A volume of 20 μL of this diluted gel was added to each well of a 24-well plate, and the plate was then incubated at 37°C to allow the gel to solidify for 3 hours. Following solidification, digested HUVECs were seeded into the wells at a density of 2.5 × 10^4 cells per well. The cells were cultured in a medium containing 2% fetal bovine serum (FBS) mixed with TCM in a 1:1 ratio, with a total volume of 200 μL per well. To establish a chemotactic gradient, 600 μL of culture medium enriched with 20% FBS was added to the lower chamber of the 24-well plate. The invasive potential of the HUVECs was evaluated after a 48-hour incubation in a controlled-temperature incubator.

Quantitative real-time RT-PCR

Total cellular RNA was extracted from the samples using the NCM RNA Extraction Kit (NCM Biotech, M5105) as per the manufacturer's guidelines. Subsequently, the RNA was reverse transcribed into complementary DNA (cDNA) using the HiScript II Q RT SuperMix for qPCR (+gDNA Wiper) (Vazyme, R223-01). Following cDNA synthesis, quantitative real-time polymerase chain reaction (qRT-PCR) was conducted with the HiScript II Q RT SuperMix (Vazyme, R222-01) according to the manufacturer's protocol. The specific primer sequences used were as follows: for β-actin, forward (F5′-ATTCCTATGTGGGCGACGAG-3′ and R 5′-TAGCACAGCCTGGATAGCAA-3′); for POFUT2, (F 5′-GCAGACATCTCAACTCCACG-3′ and R 5′-TCCTTTCTGACGGCATCTG-3′); for JUP, (F 5′-ACCAGCATCCTGCACAACCTCT-3′ and R 5′-GGTGATGGCATAGAACAGGACC-3′); and for VEGFA, (F 5′-CTGCTGTCTTGGGGTGCATTG-3′ and R 5′-TCACCGCCTCGGGCTTGTCACA-3′).

Western blot

Cells were lysed using RIPA lysis buffer (NCM Biotech, WB3100) on ice. Once complete lysis was achieved, protein concentrations were determined using the BCA protein assay. The protein samples were then boiled to denature them and subjected to SDS-PAGE for separation. Following electrophoresis, the proteins were transferred onto a PVDF membrane. The membrane was blocked in 5% skimmed milk for 1 hour at room temperature before incubation with the respective primary antibodies at 4 ° C overnight. After primary antibody incubation, the membrane was incubated with the corresponding secondary antibodies for 2 hours at room temperature. The immunoreactive bands were visualized using an ultrasensitive ECL chemiluminescence detection kit (NCM, P10300). The antibodies used in this study were as follows: POFUT2 (ABclonal, A12223), JUP (ABclonal, A0963), VEGFA (ABclonal, A21647; Proteintech, 19003-1-AP), GAPDH (Proteintech, 10494-1-AP), Flag (ABclonal, AE092), and AAL lectin (Vector Labs, B-1395-1).

Immunohistochemistry (IHC)

Tissue sections were first deparaffinized by baking in a thermostatic oven at 65° C for 2 hours. Subsequently, the sections were deparaffinized by sequential immersion in xylene and graded ethanol solutions (100%, 95%, 85%, 75%, and 0%) for 3 minutes each. Once dewaxing was complete, antigen retrieval was performed using a 10 × Tris-EDTA Antigen Retrieval Solution (Coolaber, SL1863). The sections were then permeabilized in phosphate-buffered saline (PBS) containing 0.1% Triton X-100 for 15 minutes and treated with an endogenous peroxidase blocking buffer (Beyotime, P0100B) for 10 minutes. Following this, the sections were incubated with an immunostaining blocking buffer (Beyotime, P0260) for an additional 10 minutes to reduce non-specific binding.The sections were then incubated with the primary antibody overnight

at 4°C, using the same primary antibodies as mentioned earlier. After primary antibody incubation, the sections were incubated with biotinylated goat anti-rabbit IgG (Beyotime, A0279) for 2 hours. This was followed by incubation with a DAB staining kit (ZSGB-BIO, ZLI-9018) for 10 minutes to visualize the immunoreactive sites. Finally, the sections were dehydrated, mounted, and the staining intensity and area were subsequently scored using ImageJ software (developed in Maryland, USA). The stained area score was: < 25% stained area was defined as 1 point, 25% < Stained area < 50% was defined as 2 points, 50% < Stained area < 75% was defined as 3 points, and stained area > 75% was defined as 4 points. Staining intensity was scored as: Negative was defined as 0, Low Positive as 1, and Positive as 2. IHC score = Staining area score * staining intensity score

Liquid Chromatography-Tandem Mass Spectrometry

Protein samples, after processing, were subjected to separation via SDS-PAGE electrophoresis. The resolved gels were subsequently stained with Thomas Brilliant Blue to visualize the protein bands. The bands of interest were carefully excised from the gel and subjected to in-depth proteomic analysis using liquid chromatography-tandem mass spectrometry (LC-MS/MS) at APTBIO.

Immunoprecipitation and lectin blotting experiment

Cells were lysed using an IP lysis buffer (NCM Biotech, P70100), and protein samples were collected by centrifugation. To each tube, 20 μl of magnetic beads were added for a 3-hour incubation to facilitate protein cross-linking. Afterward, the excess beads were then washed with the appropriate buffer and 5 μl of the corresponding primary antibody was added to each tube. For the IgG control group, 5 μl of IgG antibody (Santa Cruz Biotechnology, SC-66931)

was added.The samples were then incubated at 4°C overnight to allow for antibody binding. Following the incubation, the IP lysis buffer was used to wash the beads eight times, with each wash lasting for 5 minutes. The proteins that were bound to the beads were subsequently eluted and denatured in preparation for Western blot analysis.For the Western blot procedure, the membrane was first blocked to prevent non-specific binding. It was then incubated with AAL lectin(Vector Labs, B-1395-1) at 4°C overnight. After primary antibody incubation, the membrane was incubated with biotinylated goat anti-rabbit IgG (Beyotime, A0279) for 2 hours. Finally, the membrane was incubated with a DAB staining kit (ZSGB-BIO, ZLI-9018) for 10 minutes to visualize the staining results.

Statistical analyses

We retrieved RNA-sequencing data from 521 cases of TCGA CRC cohort from the TCGA database (https://portal.gdc.cancer.gov/). This dataset encompassed sequencing data from both cancerous and paired normal tissues across 521 patients, which were utilized for subsequent bioinformatics analysis. Employing R software, we conducted a series of analyses, including differential gene expression, Lasso regression, univariate and multivariate Cox proportional hazards regression, Kaplan-Meier survival curves, and receiver operating characteristic (ROC) curves. For statistical processing of experimental data, we utilized GraphPad Prism version 9.0.0.To ensure reproducibility, RT-PCR, CCK-8 proliferation assays, tubule formation assays, and Transwell invasion experiments were each performed in triplicate. Data are presented as the mean ± standard deviation (SD). For statistical analysis, independent samples t-tests and one-way ANOVA were employed.P-values < 0.05 were considered statistically significant differences (*p < 0.05, **p < 0.01, ***p < 0.001, ****p < 0.0001)
